# First clinical case report of *Cytauxzoon* sp. infection in a domestic cat in France

**DOI:** 10.1186/s12917-017-1009-4

**Published:** 2017-03-29

**Authors:** Jean-Pierre Legroux, Lénaïg Halos, Magalie René-Martellet, Marielle Servonnet, Jean-Luc Pingret, Gilles Bourdoiseau, Gad Baneth, Luc Chabanne

**Affiliations:** 1 Clinique vétérinaire La Toison d’Or, Dijon, France; 2 Merial, Lyon, France; 3 University of Lyon, VetAgro Sup – Veterinary Campus of Lyon, Marcy l’Etoile, France; 4 EPIA (Epidémiologie animale Unit), INRA, Saint Genès Champanelle, France; 5 Scanelis, Colomiers, France; 6 Koret School of Veterinary Medicine, Hebrew University, Rehovot, Israel

**Keywords:** Case report, *Cytauxzoon* sp., Cat, Feline piroplasmosis, Acute febrile illness, Persistent parasitemia

## Abstract

**Background:**

Feline cytauxzoonosis is an emerging infection caused by tick-transmitted apicomplexan parasites of the genus *Cytauxzoon*. The association of clinical disease with *Cytauxzoon* infection appears to be limited to *C. felis* infections in the Americas. Sporadic infections of wild and domestic felids with *Cytauxzoon* sp. were recently described in European countries but clinical reports of the infection are rare and incomplete. This case report brings new interesting information on cytauxzoonosis expression in Europe.

**Case presentation:**

A 9-years-old castrated European shorthair cat living in rural area of north-eastern France (Saint Sauveur, Bourgogne-Franche-Comté region), without any travel history was presented for consultation due to hyperthermia, anorexia, depression and prolonged fever that didn’t respond to antibiotic therapy. The cat had outdoor access with a history of vagrancy and was adequately vaccinated (core vaccines and FeLV vaccine). During biological investigations, intraerythrocytic inclusions were observed on blood smear and were further investigated by PCR analysis and sequencing. Molecular analyses confirmed *Cytauxzoon* sp. infection. The cat was treated with a subcutaneous injection of imidocarb dipropionate (3.5 mg/kg). One week after treatment, the cat improved clinically, although parasitic inclusions within erythrocytes persisted, and only a mild lymphocytosis was found. Two weeks after treatment, the cat appeared in excellent health, appetite was normal and parasitemia was negative. However, one month after treatment the cat relapsed with hyperthermia, anorexia, and depression. Blood smears and PCR were once again positive. Subsequently, the cat received an additional dose of imidocarb dipropionate (3.5 mg/kg SC) and recovered rapidly without other clinical signs. Two weeks after the second imidocarb injection, the cat was hit by a car and died.

**Conclusion:**

This case provides the first clinical description of infection by *Cytauxzoon* sp. in a domestic cat in France. These findings support the fact that cytauxzoonosis should be considered in the differential diagnosis of acute febrile illness which does not respond to antibiotic in cats with outdoor access especially in areas where populations of wild felids are present.

## Background

Feline cytauxzoonosis is an emerging infectious disease with an expanding geographic distribution caused by tick-borne apicomplexan parasites of the genus *Cytauxzoon*. Cytauxzoonosis was first identified in 1973 as the cause of mortality in domestic cats in Missouri, USA [1].

*Cytauxzoon* or *Cytauxzoon*-like parasites have been reported in various domestic and wild felids throughout the World [2, 3]. *Cytauxzoon felis* is considered as the main agent of the disease in domestic cats, mostly described in USA. *Dermacentor variabilis* and *Amblyomma americanum* have been shown to be the tick vectors of this pathogen [4, 5]. *C. felis* is responsible for a severe, often fatal disease associating clinical signs such as anemia, depression, anorexia, vomiting, icterus, splenomegaly, hepatomegaly and high fever [6].

However, with rare exceptions, the recognition of clinical disease caused by parasites of the genus *Cytauxzoon* appears limited to *C. felis* infections in the Americas.

Sporadic infections of wild and domestic felids with a new and genetically distinct species described as “*Cytauxzoon* sp.” were reported in European countries such as Spain [7–11], France [11], Italy [12–14], Portugal [15], and Romania [16], but reports presenting clinical expression of the infection are rare and incomplete. *Cytauxzoon* sp. in Europe seems less virulent than *C. felis* and it was suggested that disease associated with it could develop preferentially in case of concurrent disease or immunodeficiency [12]. Hemolytic anemia, lethargy, fever, anorexia, weight loss, diarrhea and vomiting were occasionally described in association with *Cytauxzoon* sp*.* infection [12, 13, 15]. A high rate of asymptomatic carriage of *Cytauxzoon* sp. in domestic cats was reported in a study conducted in Northern Italy [12]. In France, a previous study reported the case of a cat found co-infected with *Hepatozoon canis* and *Cytauxzoon* sp. The agents were molecularly characterized but no information on the epidemiology, clinical history, clinical course of infection and therapy was available [11]. Consequently, knowledge on the epidemiology, risk factors and clinical course of infection of felids with *Cytauxzoon* sp. in European countries and in particular in France is still unclear and needs further investigation. This study provides the first clinical description of infection by *Cytauxzoon* sp. in a domestic cat in France.

## Case presentation

### Clinical history

A 9-years-old neutered male European shorthair cat weighting 6 kg and living in a rural area (Saint Sauveur, France: 47°48′N, 6°23′E), without any travel history was presented to consultation for lethargy, anorexia, hemorrhagic diarrhea, and abdominal pain. The cat had permanent outdoor access and was vaccinated against feline panleukopenia, viral rhinotracheitis, calicivirus and feline leukemia virus.

Two weeks before consultation, the cat came back from 4 days vagrancy with hyperthermia (41 °C), lethargy, anorexia, dehydration and weight loss. The cat didn’t recover within the following 15 days despite antibiotic therapy of amoxicillin and clavulanic acid at 10 mg and 2.5 mg/kg respectively (Clavaseptin, Vetoquinol) administered orally every 12 h for 10 days.

### Clinical findings and investigation (Table 1)

Clinical examination showed hyperthermia (40.5 °C), abdominal pain and subcutaneous hematomas on the abdomen. Sepsis resulting from a possible fight during vagrancy was suspected and subcutaneous injections of marbofloxacin (Marbocyl, Vetoquinol, 2 mg/kg) and carprofen (Rimadyl, Zoetis, 4 mg/kg) were performed followed by marbofloxacin (Marbocyl, Vetoquinol) 2 mg/kg PO once daily for 10 days and meloxicam (Metacam, Boehringer Ingelheim) at 0.05 mg/kg PO once daily for 5 days for pain and fever relief.

**Table 1:** Timeline table of the information of the case report

Day (D)	Findings		Treatment
	Clinical observation	Clinical investigation	
D-14	Clinical history: hyperthermia (41 °C), lethargy, anorexia, dehydration and weight loss after 4 days vagrancy		amoxicillin (10 mg/kg) + clavulanic acid (2.5 mg/kg) orally every 12 h for 10 days.
D0	hyperthermia (40.5 °C), abdominal pain and subcutaneous hematomas on the abdomen		marbofloxacin (2 mg/kg) and carprofen (4 mg/kg) marbofloxacin (2 mg/kg PO once daily for 10 days meloxicam 0.05 mg/kg PO once daily for 5 days
D7 to D29		neutrophilic leukocytosis at the complete blood counts (CBC), biochemical, serological and molecular analyses for different infectious disease: all negative Abdominal, pulmonary and oral X-ray: normal except a marked splenomegaly oropharyngeal swab for the detection of calicivirus and feline herpes virus were conducted	
D29	no clinical improvement vomiting, stomatitis, abdomen pain, hyperthermia, anorexia, severe depression	Stained blood smear of peripheral blood: small inclusions within erythrocytes PCR identification of *Cytauxzoon* sp.	imidocarb dipropionate 3.5 mg/kg/ subcutaneous injection).
Day 37	Clinical improvement	Inclusions present in the blood smear	
Day 46	Excellent general conditions	No more inclusions in the blood smear	
Day 63	Relapse: hyperthermia, anorexia depression and stomatitis	Inclusions present in the blood smear PCR positive for *Cytauxzoon* sp.	imidocarb dipropionate 3.5 mg/kg/ subcutaneous injection).
Day 69	Clinical recovery	hypergammaglobulinemia with hyperproteinemia (91 g/L; reference range: 57 – 94 g/L) with an hematocrit at the lower limit of the reference range (28 L/L; reference range: 28 – 45). Absence of inclusion in the blood smear	
Day 76	Death by car accident		

The cat was rechecked 29 days later. The owner reported no clinical improvement despite proper administration of the treatment and subsequent vomiting, stomatitis, cranial right abdomen pain, hyperthermia, anorexia and severe depression were noticed. The cat was hospitalized and received symptomatic treatment against vomiting (maropitant, Cerenia, Zoetis, SC injection, 1 mg/kg) as well as a corticosteroid (dexamethasone tebutate, Dexamedium, MSD, SC injection, 0.1 mg/kg) and antibiotics (amoxicillin, Duphamox LA, Zoetis, SC injection, 15 mg/kg every other day for 1 week, followed by cefovecine, Convenia, Zoetis, SC injection, 8 mg/kg).

Blood samplings were performed on days 7, 18, 21 and 29 for complete blood counts (CBC), and/or biochemical, serological and molecular analyses of common infectious diseases. Abdominal, pulmonary and oral X-ray with oropharyngeal swab for the detection of calicivirus and feline herpes virus were conducted.

### Differential diagnosis

The rapid immune-migration test for feline leukemia virus and feline immunodeficiency virus diagnosis (Witness®, Synbiotics corp.), Coombs test and PCR for the detection of feline hemotropic mycoplasms (*Mycoplasma haemofelis* and *Candidatus* *Mycoplasma haemominutum*) were all negatives. PCR detection of calicivirus and feline herpes virus on the oropharyngeal swab were also negative and the X-rays were interpreted as normal with the exception of a marked splenomegaly.

The CBC performed on day 7 (Table 2) showed red blood cells count within normal limits, an increased white blood cells count (WBC: 25.3 × 10^9^/L; reference range: 5 – 11) with neutrophilic leucocytosis (neutrophils: 17.7 × 10^9^/L, confirmed on blood smear with relatively frequent Döhle inclusion bodies; reference range: 3 – 11), and eosinophilia (eosinophils: 1.8 × 10^9^/L; reference range: 0 – 0.6).

**Table 2:** Summary of blood cell counts at the time of diagnosis and at the last recheck before the cat’s death with maximum and minimum values observed during the 78 days of follow-up

Parameter (unit)	Reference interval	Initial analysis (day 7)	Last analysis (day 69)	Minimum and maximum value during follow-up
RBC^a^ (10^12^/L)	5 – 10	8.00	8.73	6.75 – 8.73
Hemoglobin (g/L)	90 – 150	122	115	102 – 115
Hematocrit (L/L)	28 – 45	36.0	28	28 – 37.1
MCV^b^ (fL)	40 – 55	45	32	32 – 47
MCHC^c^ (g/L)	310 – 350	339	410	280 – 345
WBC^d^ (10^9^/L)	5 – 11	25.3	15.0	11 – 12.8
Neutrophils (10^9^/L)	3 – 11	17.7	7.6	5.4 – 9.25
Eosinophils (10^9^/L)	0 – 0.6	1.8	0.95	0.15 – 0.5
Monocytes (10^9^/L)	0 – 0.5	0.500	0.55	0.15–1.85
Lymphocytes (10^9^/L)	1 – 6	5.3	5.9	3–6.5
Platelets (10^9^/L)	150 – 550	165	305	305 – 441

^a^ *RBC* red blood cells count

^b^ *MCV* mean corpuscular volume

^c^ *MCHC* mean corpuscular hemoglobin concentration

^d^ *WBC* white blood cells count

Serum biochemistry indicated an hypertriglyceridemia (7.14 mmol/L; reference range: 0.13 – 1.61) whereas all other biochemical parameters tested including urea, creatinine, glucose, ALT, ALKP, and lipase were within normal limits.

**Fig. 1:**
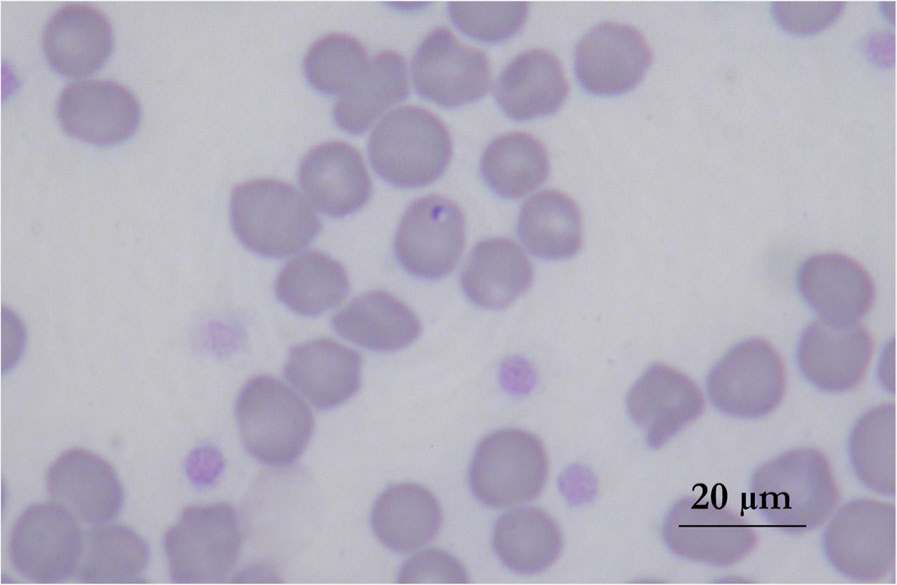
Stained blood smear showing *Cytauxzoon* sp. parasite in cat erythrocyte

The stained blood smear of peripheral blood examined on day 29 revealed small inclusions within erythrocytes (Fig. 1) with uniform distribution on the smear (one red blood cell inclusion for two fields at high magnification: ×1000). The inclusions had an annular shape and were 0.5-0.8 μm in diameter suggesting the possibility of infection with and unidentified small piroplasmid parasite. A marked agglutination of erythrocytes was also noted on slide. No schizont-infected myeloid cells were detected. The blood smears done previously (on day 7, 18 and 21) were not available to be re-assessed.

### Final diagnosis

**Fig. 2:**
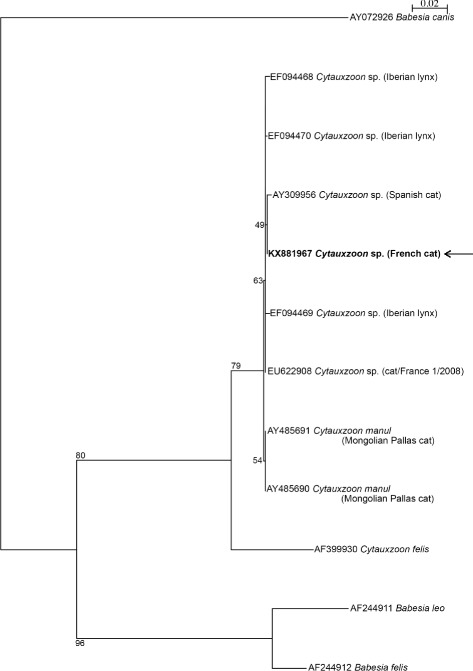
Phylogenetic analysis based on 944-bp of 18S rDNA sequences of *Babesia* and *Cytauxzoon* species infecting wild and domestic Felids. Consensus sequence from the infected cat described in the present case report is indicated by an arrow. Identity and Genbank® accession numbers are indicated for each sample. The phylogenetic tree was constructed using the Hasegawa, Kishino and Yano maximum likelihood method, with bootstrap analysis with 1000 replicates. The tree was rooted using *Babesia canis* as an outgroup. Subsequent analyses using the Kimura’s two-parameter (K2P) distance and the parsimony methods in the same conditions confirmed the topology of the tree

DNA was extracted from whole blood collected in EDTA on day 29 and was simultaneously analyzed by PCRs on the 18S rRNA gene of the Piroplasmida in three different laboratories (Scanelis laboratory, Toulouse, France; Laboratory of Parasitology and Parasitic diseases, Vetagro Sup, Marcy-l’Etoile, France; and Koret School of Veterinary Medicine laboratory at the Hebrew University) following the conditions summarized in Table 3 [17, 18]. PCR was positive in all laboratories and the amplified DNA was sequenced. The three overlapping sequences of 18S rDNA fragments obtained from each laboratory were 100% homologous to each other in the common fragments. A consensus sequence of 944 bp was deposited in Genbank under accession number KX881967. Comparison of this consensus sequence to sequences deposited in GenBank® using the basin local alignment search tool (BLAST) (https://blast.ncbi.nlm.nih.gov/Blast.cgi) as well as phylogenetic analyses (Fig. 2) affiliated the organism found in the cat’s blood to *Cytauxzoon* sp. with 99% identity with sequences of *Cytauxzoon* sp. previously detected from domestic cats in France (EU622908) and Spain (AY309956) and from wild lynxes in Spain (EF094468 to EF094473). It also had a 99% identity with *Cytauxzoon manul* from Mongolian wild cats (AY485690 and AY485691).

**Table 3:** List of the primers used for the amplification of 3 overlapping fragments of 18S rDNA of piroplasms

Primer names	Primers sequences	Product size bp	PCR conditions	Reference
Nested PCR: BTF1(external) BTR1 (external)	GGCTCATTACAACAGTTATAG CCCAAAGACTTTGATTTCTCTC	930 bp	94 °C 3 min, 58 °C 1 min 72 °C 2 min 45 cycles: 94 °C 30s, 58 °C 20s 72 °C 30s 72 °C 7 min	[17]
BTF2 (internal) BTR2 (internal)	CCGTGCTAATTGTAGGGCTAATAC GGACTACGACGGTATCTGATCG	836 bp	Same conditions for the secondary round with an annealing temperature of 62 °C	
Paraseq1F_scanelis 18seq1R_scanelis	TGGCTCATTAMAACAGTTATAGTTTA AGACAAATCRCTCCACCAAC	1188	94 °C 3 min 45 cycles: 94 °C 20 s 56 °C 30 s 72 °C 45 s 72 °C 7 min	Unpublished
Piro-A Piro-B	AAT ACC CAA TCC TGACAC AGG G TTA AAT ACG AAT GCC CCC AAC	408 bp	94 °C 1 min 39 cycles 94 °C 45 s, 62 °C 45 s, 72 °C 45 s. 72 °C 7 min	[18]

### Treatment, outcome and follow-up

On day 29, the cat was treated with a subcutaneous injection of imidocarb dipropionate (Carbesia, MSD, 3.5 mg/kg).

One week after treatment (day 37), the cat improved clinically, however, intraerythrocytic inclusions were still present, and a mild lymphocytosis was noticed on the blood smear.

Two weeks after treatment (day 46), the cat appeared in excellent health, appetite was normal and parasitemia was negative by blood smear microscopic examination.

However, one month after treatment (day 63) the cat relapsed and was presented with a new episode of hyperthermia, anorexia depression and stomatitis. CBC was within the normal values, but intraerythrocytic inclusions were again present on the blood smears and PCR was positive. The cat received an additional injection of imidocarb dipropionate (3.5 mg/kg SC) and recovered rapidly without other clinical signs. An extended CBC and a serum protein analysis performed 7 days later (on day 69) indicated an hypergammaglobulinemia with hyperproteinemia (91 g/L; reference range: 57 – 94 g/L) with an hematocrit at the lower limit of the reference range (28 L/L; reference range: 28 – 45). Oropharyngeal examination was normal.

Unfortunately, two weeks after the second imidocarb injection (day 76), the cat was hit by a car and died. This didn’t allow further clinical investigations.

### Post-mortem examination

No specific macroscopic lesions related to the cat’s disease were found on gross pathology. *Post-mortem* samples from the liver, spleen, kidney, iliac lymph node, lung and bone marrow as well as smears from the lung, spleen and marrow were collected and sent to the laboratory of Histology, VetAgro Sup, Marcy l’Etoile, France, for cytology and histology. *Cytauxzoon* schizonts were not encountered in the macrophages of any tissue. Layers from lungs showed suspicious inclusions in macrophages that could not be more precisely characterized. The histopathological findings observed included hyperplasia of the pancreas and of the spleen as well as an extended renal cortical inflammatory lesion. Changes in the lungs tissues related to the car crash were also noticed.

## Discussion

The present case is the first report of a relapsing fever responsive to treatment with imidocarb dipropionate associated with *Cytauxzoon* sp. infection in a cat in France. It describes the course of the infection and brings new information about clinical expression of suspected cytauxzoonosis in the domestic cat in Europe.

The clinical signs reported here correspond to the signs previously described during infection with *Cytauxzoon* sp. (and usually described in case of piroplasms infection in other species). The facts that (i) no other cause (infectious in particular) could be evidenced, that (ii) intra-erythrocytic inclusions were systematically detected in acute phases of the disease and that (iii) clinical improvement was noticed after treatment with imidocarb dipropionate, highly comfort the role of *Cytauxzoon* sp. in the clinical signs observed. However it is not possible to ascertain that no other concurrent disease was present. Experimental infection should be conduct in the future to assess the clinical expression of European *Cytauxzoon* sp. infection.

Based upon results from the sequencing and the phylogenetic analyses performed on the 18S ribosomal RNA genes, *Cytauxzoon* sp. detected in this clinical case presented a high homology (99%) with *Cytauxzoon* sp. and *Cytauxzoon manul* previously detected in wild and domestic felids from Europe and Mongolia [7, 9, 11, 19]. In contrast, the sequence revealed an identity of only 95% with *C. felis* sequences deposited in GenBank®. The parasites described from Europe as *Cytauxzoo*n sp. are closely related and may belong to the same species. They are distinct from *Cytauxzoon felis,* the agent of feline cytauxzoonosis in the New World.

Epidemiological and clinical data on *Cytauxzoon* sp*.* infection in domestic and wild felids in Europe have rarely been reported and originate from Spain, Italy, France, Portugal and Romania [7, 9–13, 15, 16].

In the domestic cat, single occasional molecular detections of *Cytauxzoon* sp. have been described in France [11] and Spain [11] with no associated clinical finding. More recently, widespread *Cytauxzoon* sp. infection (up to 23% prevalence by PCR) have been documented in a population of domestic cats living in Trieste in North-Eastern Italy [12]. Infection was sub-clinical in the majority of infected cats, however, clinical signs were described in 3 cats. Additional clinical cases have been reported in 2 young cats from Central Italy [13] and in a domestic cat from Portugal [15]. The clinical signs and history reported for Italian cats [12, 13], included gastro-intestinal disorders (vomiting, diarrhea), weight loss, hyperthermia (>40 °C), stomatitis, ulcerative dermatitis, and lethargy. Persistent parasitemia was also described [12]. A similar clinical pattern was observed in the present case characterized by nonspecific signs of acute febrile illness including lethargy and hyperthermia without a marked impact on the CBC and serum biochemical parameters. Stomatitis and persistent parasitemia despite clinical improvement were also described. This pattern appears to differ in severity compared to what is described in the US. Cytauxzoonosis due to *C. felis* is characterized by an acute febrile illness with profound fever (although hypothermia may be identified in moribund cats), depression and vocalization (so-called death yowl). Laboratory abnormalities at the time of presentation are more marked and include frequent thrombocytopenia and neutropenia as well as non-regenerative anemia, leukopenia, hyperbilirubinemia and bilirubinuria, mild elevations in liver enzymes, hyperglycemia and hypoalbuminemia [2, 3]. Interestingly, the Portuguese case was evocative of the American disease. The cat displayed a panel of signs compatible with a severe hemolytic disease including anemia, leukocytosis, thrombocytopenia and azotemia [15]. However, genetic analyses clustered the Portuguese sequence with European *Cytauxzoon* sp. [15].

Supportive and critical treatment is a mainstay of therapy for American cytauxzoonosis [2, 3, 20]. Clinical recovery is not rapid with most patients getting worse during the first 24-48 h followed by a gradual improvement over the next several days. A randomized clinical trial demonstrated survival rates of 60% with a combination of atovaquone and azithromycin compared to 27% with imidocarb dipropionate [21]. In the Italian studies, treatments were based on different combinations of antiprotozoal drugs with antibiotics and steroids. One cat received a combination of imidocarb dipropionate and enrofloxacin 15 days apart followed by doxycycline, azithromycin and atovaquone without clinical improvement and was eventually euthanized. A second was treated with azithromycin, enrofloxacin and prednisone and survived, while a third cat received a single administration of azithromycin and imidocarb dipropionate and was euthanized in the absence of improvement [12]. The two other cats [13] were initially treated with doxycycline, followed by imidocarb dipropionate injections (5 mg/kg, IM injection, for two times 2 weeks apart). The 2 cats improved during a follow-up period of 175 days and 130 days respectively, and the treatment seemed to eliminate *Cytauxzoon* sp. infection. The Portuguese cat received daily administration of azithromycin (10 mg/kg) alone, and the animal died despite supportive care [15].

In our case, it is difficult to assert the clinical improvement as the cat accidentally died after a relapse and a second treatment with imidocarb dipropionate at a monthly interval.

Interestingly, detection of schizont-infected white blood cells on the initial blood smear wasn’t noticed in any of the European cases, including the present one. In the US, the identification of schizont-infected myeloid cells confirms acute cytauxzoonosis due to *C. felis*. The absence of detection of schizonts may be related to a difference in the schizogony of the species present in Europe. This would need further investigation. No schizont-infected cells were either identified at necropsy.

Infection with *Cytauxzoon* sp. is usually associated with an outdoor life style. This is probably consistent with the suspected tick-borne transmission mode of this infection. The vector species of cytauxzoonosis in Europe is not identified yet. Interestingly, as for the Italian study of Carli et al. in 2012 [12], the cat in our report originated from an area close to the region where the presence of Eurasian lynx (*Lynx lynx*) was documented and where wild cats (*Felis silvestris*) are also present. *Cytauxzoon* sp. was also detected in Iberian lynxes (*Lynx pardinus*) from southern Spain [9, 10] suggesting a circulation of this parasite in wild and domestic felid populations of given areas. Additional studies are needed to understand the biology of the European species of *Cytauxzoon* and their distribution in European countries.

## Conclusion

This case provides the first clinical description of disease associated with *Cytauxzoon* sp. in a domestic cat in France. While further investigations are needed to understand the relationship of the European strains of *Cytauxzoon* with the observed symptoms, these findings support the fact that cytauxzoonosis should be considered in the differential diagnosis of acute fever not responsive to antibiotic treatment in cats with outdoor access, especially in areas where populations of wild felids are present.

## References

[1] Wagner JE (1976). A fatal cytauxzoonosis-like disease in cats. J Am Vet Med Assoc.

[2] Cohn LA, Birkenheuer AJ, Greene CE (2012). Cytauxzoonosis. Infectious diseases of the dog and cat.

[3] Lloret A, Addie DD, Boucraut-Baralon C, Egberink H, Frymus T, Gruffydd-Jones T, Hartmann K, Horzinek MC, Hosie MJ, Lutz H, Marsilio F, Pennisi MG, Radford AD, Thiry E, Truyen U, Möstl K, European Advisory Board on Cat Diseases. Cytauxzoonosis in cats (2015). ABCD guidelines on prevention and management. J Feline Med Surg..

[4] Blouin EF, Kocan AA, Glenn BL, Kocan KM, Hair JA (1984). Transmission of *Cytauxzoon felis* kier, 1979 from bobcats, *Felis rufus* (Schreber), to domestic cats by *Dermacentor variabilis* (say). J Wildl Dis.

[5] Reichard MV, Edwards AC, Meinkoth JH, Snider TA, Meinkoth KR, Heinz RE, Little SE (2010). Confirmation of *Amblyomma americanum* (Acari: Ixodidae) as a vector for *Cytauxzoon felis* (Piroplasmorida: Theileriidae) to domestic cats. J Med Entomol.

[6] Birkenheuer AJ, Le JA, Valenzisi AM, Tucker MD, Levy MG, Breitschwerdt EB (2006). Cytauxzoon felis infection in cats in the mid-Atlantic states: 34 cases (1998-2004). J Am Vet Med Assoc.

[7] Criado-Fornelio A, Gónzalez-del-Río MA, Buling-Saraña A, Barba-Carretero JC (2004). The “expanding universe” of piroplasms. Vet Parasitol.

[8] Luaces I, Aguirre E, García-Montijano M, Velarde J, Tesouro MA, Sánchez C, Galka M, Fernández P, Sainz A (2005). First report of an intraerythrocytic small piroplasm in wild Iberian lynx (*Lynx pardinus*). J Wildl Dis.

[9] Millán J, Naranjo V, Rodríguez A, de la Lastra JM, Mangold AJ, de la Fuente J (2007). Prevalence of infection and 18S rRNA gene sequences of Cytauxzoon species in Iberian lynx (*Lynx pardinus*) in Spain. Parasitology.

[10] Millán J, Candela MG, Palomares F, Cubero MJ, Rodríguez A, Barral M, de la Fuente J, Almería S, León-Vizcaíno L (2009). Disease threats to the endangered Iberian lynx (*Lynx pardinus*). Vet J.

[11] Criado-Fornelio A, Buling A, Pingret JL, Etievant M, Boucraut-Baralon C, Alongi A, Agnone A, Torina A (2009). Hemoprotozoa of domestic animals in France: prevalence and molecular characterization. Vet Parasitol.

[12] Carli E, Trotta M, Chinelli R, Drigo M, Sinigoi L, Tosolini P, Furlanello T, Millotti A, Caldin M, Solano-Gallego L (2012). *Cytauxzoon* sp. infection in the first endemic focus described in domestic cats in Europe. Vet Parasitol.

[13] Carli E, Trotta M, Bianchi E, Furlanello T, Caldin M, Pietrobelli M, Solano-Gallego L (2014). *Cytauxzoon* sp. infection in two free ranging young cats: clinicopathological findings, therapy and follow up. Turkiye Parazitol Derg.

[14] Veronesi F, Ravagnan S, Cerquetella M, Carli E, Olivieri E, Santoro A, Pesaro S, Berardi S, Rossi G, Ragni B, Beraldo P, Capelli G (2016). First detection of *Cytauxzoon* spp. infection in European wildcats (*Felis silvestris silvestris*) of Italy. Ticks Tick Borne Dis.

[15] Alho AM, Silva J, Fonseca MJ, Santos F, Nunes C, de Carvalho LM, Rodrigues M, Cardoso L (2016). First report of *Cytauxzoon* sp. infection in a domestic cat from Portugal. Parasit Vectors.

[16] Gallusová M, Jirsová D, Mihalca AD, Gherman CM, D'Amico G, Qablan MA, Modrý D (2016). *Cytauxzoon* infections in wild felids from Carpathian-Danubian-Pontic space: further evidence for a different *Cytauxzoon* species in European felids. J Parasitol.

[17] Jefferies R, Ryan UM, Irwin PJ (2007). PCR-RFLP for the detection and differentiation of the canine piroplasm species and its use with filter paper-based technologies. Vet Parasitol.

[18] Olmeda AS, Armstrong PM, Rosenthal BM, Valladares B, del Castillo A, de Armas F, Miguelez M, Gonzalez A, Rodriguez JA, Spielman A, Telford SR (1997). A subtropical case of human babesiosis. Acta Trop.

[19] Reichard MV, Van Den Bussche RA, Meinkoth JH, Hoover JP, Kocan AA (2005). A new species of *Cytauxzoon* from Pallas’ cats caught in Mongolia and comments on the systematics and taxonomy of piroplasmids. J Parasitol.

[20] Sherrill MK, Cohn LA (2015). Cytauxzoonosis: diagnosis and treatment of an emerging disease. J Feline Med Surg.

[21] Cohn LA, Birkenheuer AJ, Brunker JD, Ratcliff ER, Craig AW (2011). Efficacy of atovaquone and azithromycin or imidocarb dipropionate in cats with acute cytauxzoonosis. J Vet Intern Med.

